# 5-HIAA induces neprilysin to ameliorate pathophysiology and symptoms in a mouse model for Alzheimer’s disease

**DOI:** 10.1186/s40478-018-0640-z

**Published:** 2018-12-11

**Authors:** Christian Klein, Guy Roussel, Susana Brun, Cristina Rusu, Christine Patte-Mensah, Michel Maitre, Ayikoe-Guy Mensah-Nyagan

**Affiliations:** Biopathologie de la Myéline, Neuroprotection et Stratégies Thérapeutiques, INSERM U1119, Fédération de Médecine Translationnelle de Strasbourg (FMTS), Université de Strasbourg, Bâtiment 3 de la Faculté de Médecine, Strasbourg, France

**Keywords:** Alzheimer’s disease, Neprilysin, Aβ peptides, 5-HIAA, Serotonergic transmission, ERK and GSK-3 pathways

## Abstract

Serotoninergic activation which decreases brain Aβ peptides is considered beneficial in mouse models for Alzheimer’s disease (AD), but the mechanisms involved remain unclear. Because growing evidence suggested that the stimulation of proteases digesting Aβ, especially the endopeptidase neprilysin (NEP) may be effective for AD therapy/prevention, we explored the involvement of serotonin precursors and derivatives in NEP regulation. We found that 5-hydroxyindolacetic acid (5-HIAA), the final metabolite of serotonin, considered until now as a dead-end and inactive product of serotonin catabolism, significantly reduces brain Aβ in the transgenic APPSWE mouse model for AD-related Aβ pathology and in the phosphoramidon-induced cerebral NEP inhibition mouse model. 5-HIAA treatment improves memory performance in APPSWE mice. Furthermore, 5-HIAA and its precursors increase NEP level in vivo and in neuroblastoma cells. Inhibition of ERK 1/2 cascade by 5-HIAA or SCH772984 enhanced NEP levels, suggesting MAP-kinase pathway involvement in 5-HIAA-induced regulation of NEP expression. Our results provide the first demonstration that 5-HIAA is an active serotonin metabolite that increases brain Aβ degradation/clearance and improves symptoms in the APPSWE mouse model for AD.

## Introduction

Alzheimer’s disease (AD) is a multifactorial neurodegenerative disorder resulting from proteinopathies characterized by the accumulation/ aggregation of β amyloid peptides (Aβ) and hyperphosphorylation followed by aggregation of microtubule-associated protein Tau [48, 52]. Before the symptomatic expression of the disease, a long incubation time over decades is characterized by microinflammation, vasculopathy, metabolic disturbances that favorize the appearance of neurodegenerative processes in aged population. Alterations of the genes encoding amyloid precursor protein (APP), presenilin-1 or 2 (PS1 or PS2), Adamalysin 10 or Apolipoprotein E4 (APOE4) are frequent in familial AD [12], but the large majority of AD patients show increased brain levels of neurotoxic Aβ without changes of the gene aforementioned. In aged patients, it is postulated that various accumulated mutations determine or favor deleterious proteinopathies, the most frequent being an anomaly in the synthesis or elimination of amyloid peptides [41]. This in turn initiates a cascade of post-translational disturbances, including mainly the hyperphosphorylation and accumulation of Tau. These events produce an early symptomatology made of significant memory and cognitive impairment, generally accompanied by measurable brain atrophy due to neurodegeneration [49].

Several approaches have been proposed to limit Aβ synthesis or to increase their elimination [2]. To reach this goal, the potentiation of some protease activities able to disaggregate pathologic Aβ substrates has been studied since several years. Among possible proteolytic enzymes, the most promising seems to be neprilysin (NEP) [39]. This enzyme belongs to the family of human M13 zinc-dependent endopeptidases which cleave a wide spectrum of brain regulatory peptides, including Aβ peptides [36]. Hyper-expression of NEP gene in the brain of transgenic mouse models for AD decreases the incidence of amyloid pathology, and NEP activity is age dependently reduced in the rodent and human brain [2].

Besides the genetic manipulation of NEP expression, pharmacological approaches used to modulate NEP activity revealed that somatostatin and various polyphenols up-regulate NEP activity in the brain [44]. Epigenetic mechanisms via HDAC inhibition also seem to be a strategy to potentiate brain NEP expression and to protect against AD progression [35]. Valproic acid and gamma-hydroxybutyrate for example have been reported to decrease brain Aβ concentration, ameliorating in parallel memory performance in mouse models for AD [28, 51].

In this context, it is known that several metabolites of tryptophan pathways through kynurenine or serotonin metabolism are implicated in neuroprotection, regulation of brain Aβ levels, excitotoxicity, cognitive and synaptic maintenance [30]. Thus, several metabolites of kynurenine and serotonin pathways are suspected to be involved in AD-related neurodegenerative processes. However, the balance between these two pathways seems to be regulated by numerous factors among which neuroinflammation may promote the kynurenine pathway and in parallel impact serotonin synthesis. Because several indications favor a role of SSRI and serotonin receptors in AD pathophysiology and progression in human and animal models [24], we examined the possible interference of serotonin intermediates on brain Aβ clearance and evidenced a specific role for 5-HIAA which is generally thought to be a dead-end and inactive product of serotonin catabolism. To strengthen our investigations and consolidate our findings, we have used two different experimental models, namely the transgenic APPSWE mouse model for AD-related Aβ pathology (Tg2576) and the mouse model of phosphoramidon-evoked brain NEP inhibition [17, 20–22, 54].

## Materials and methods

### In vitro experiments

#### Cell culture

Human neuroblastoma SH-SY5Y cells and human neuroblastoma SH-SY5Y-APPwt cells stably transfected with DNA constructs harboring human wild-type APP695 (APPwt) were maintained in a humidified atmosphere of 95% air and 5% CO_2_ at 37 °C [28]. Cells were seeded into plates or 100 mm dishes in Dulbecco modified Eagle medium, supplemented with 10% (vol/vol) fetal bovine serum, 100 U/mL penicillin, 100 μg/mL streptomycin (Fisher Scientific, France) and for the transfected cells with 300 μmg/mL hygromycin (Sigma-Aldrich, France), a selective antibiotic. Between 70 and 90% confluency, cells were incubated during various time periods and/or with various concentrations of compounds to be tested.

#### RNA isolation and quantitative PCR

Total RNA was extracted from SH-SY5Y cells after 0, 30, 60 and 90 min of treatment with 30 μM 5-HIAA, using the Nucleospin RNA L protocol (Macherey-Nagel, Düren, Germany). This protocol included a treatment of isolated RNA by DNase I. Integrity and purity of RNA was checked by spectrophotometry.

Reverse transcription was performed with 1 μg RNA using Biorad iScript® cDNA synthesis kit. q-PCR was performed in an iCycler thermal cycler (Biorad, Hercules, CA, USA) using SYBR Green dye (iQ SYBR green Supermix, Biorad). For each sample, the reaction mix was a make-up of 320 nM forward primer (F), 320 nM reverse primer (R), 200 nM probe, and 4 μL cDNA template in a total reaction volume of 20 μl [3]. Using the iCycler iQ optical system software (version 3.1, Bio-Rad), a standard curve based on successive cDNA dilutions was performed and was used to calculate starting quantities. To ensure a thorough calculation, starting quantities of genes of interest were reported to those of a housekeeping gene (U6 or Actin) in the same plate.

All samples were analyzed in triplicates, and the mean and standard deviation were calculated. After each q-PCR, specificity of the amplification was controlled by a melting curve ranging from 55 to 95 °C whereby a single peak corresponding to the amplicon was present.

The following primer pairs were used to amplify cDNAs after reverse transcription experiments [26]:

**Table:** 

Gene	Primer
Neprilysin	F 5’-CCTGGAGATTCATAATGGATCTTGT-3’
	R 5’-AAAGGGCCTTGCGGAAAG-3’
U6	F 5’-CTCGCTTCGGCAGCACA-3’
	R 5’-AACGCTTCACGAATTTGCGT-3’
Actin	F 5’-CGCAGCAGTCAGGGACATTT-3’
	R 5’-TTCACATACAGCTTGGGAAGC-3’

#### Measurement of neprilysin protein level

SH-SY5Y cells were washed with PBS and homogenized with lysis buffer containing a protease inhibitor cocktail (Sigma-Aldrich). The proteins in whole cell lysates were quantified using the BCA protein assay kit with BSA as a standard (Pierce, Rockford, IL, USA).

NEP protein levels were measured by using a DuoSet ELISA kit (R&D Systems Europe, Oxford, UK) according to the manufacturer’s guidelines with minor modifications. Goat anti-human NEP (1.6 mg/mL) diluted in PBS (pH 7.4) was coated overnight on a high-binding 96 well plate (R&D Systems Europe) at room temperature (RT). The plates were washed 3 times in PBS containing 0.5% Tween-20 (Sigma-Aldrich) (PBS-T). Non-specific binding of antibody was blocked by addition of PBS completed with 1% Bovine serum albumin (Sigma-Aldrich) (1% PBS-BSA) for 3 h at RT, then the plates were washed a further 3 times. Serial dilutions of recombinant human NEP or crude homogenates diluted in 1% PBS-BSA or 1% PBS-BSA alone as a control were incubated for 2 h with continuous shaking at RT. After a further 3 washes, biotinylated anti-NEP (1.6 mg/mL) was added for 2 h before another wash and incubation with streptavidin-peroxidase (1200) for 20 min in the dark. Substrate solution (tetramethyl benzidine; R&D Systems, Europe) was added for 30 min, and the optical density for each well was read at 450 nm and 540 nm or 570 nm in a plate reader (Elisa reader model Sigma 960, Metertech, Taipei, Taiwan).

The NEP protein levels were interpolated from the standard curve generated from serial dilutions of recombinant human NEP (R&D Systems Europe). Each measurement was repeated on 3 occasions, and the average value was calculated.

#### Cell treatment with precursors or inhibitors of the serotonin pathway

Different concentrations (0 to 100 μM) of 5-HIAA, 5-HTP, Serotonin hydrochloride (5-HT, Sigma-Aldrich) and Tryptophan (Trp, Sigma-Aldrich), were used. Pharmacological treatments were performed with specific inhibitors ((S)-(−)-carbidopa monohydrate (Abcam, Cambridge, UK), tranylcypromine hydrochloride (2-PCPA, Abcam), clorgyline hydrochloride (Abcam) were added to the SH-SY5Y cells 90 min or 24 h before harvest.

ERK inhibitor SCH772984 (Carbosynth, Compton Berkshire, UK), MEK ½ inhibitor GSK 1120212 (Targetmol, USA) or GSK-3 inhibitor CHIR99021 (Sigma-Aldrich, France) was added 30 min before the measurement of NEP protein.

#### SH-SY5Y-APP cell treatment and Western blot analysis

According to experimental design [26], the SH-SY5Y cells were incubated either with 10 mM ammonium chloride (NH_4_Cl, Sigma-Aldrich) or 30 μM 5-HIAA (Sigma-Aldrich) for 24 h. Non-treated cells were used as control. After 24 h of treatment, the cells were rinsed twice with cold PBS pH 7.4, harvested and pelleted by centrifugation. The cell’s pellets were lysed in RIPA buffer (10 mM Tris/HCl pH 8.0, 150 mM NaCl, 1% (vol/vol) Nonidet P-40, 0.5% (wt/vol) sodium deoxycholate, 5 mM EDTA) with complete inhibitor mix from Roche diagnostics (Basel, Switzerland) at 0 °C for 20 min. The lysates were homogenized through 21-G needles 10 times, and then clarified by centrifugation at 2800 x g for 10 min. Protein concentration of lysates was determined using a BCA protein assay kit.

A total of 50 μg proteins were fractionated by 4–20% TGX SDS polyacrylamide gels (BioRad, Hercules, CA, USA). After electrotransfer to polyvinylidene fluoride membranes (BioRad), these membranes were blocked overnight at 4 °C in Tris-buffered saline (TBS: 50 mM Tris, 150 mM NaCl) containing 0.1% (vol/vol) Tween-20 (Sigma-Aldrich) (TBS-T) and 5% (wt/vol) skimmed milk powder. Membranes were incubated 2 h at RT with primary antibodies, Rabbit antibody against anti-APP C-terminal fragment (APP intracellular domain, AICD, Sigma-Aldrich) and Mouse monoclonal anti-β-actin antibody (Sigma-Aldrich) (1: 1000 and 1: 5000, respectively). After washing 3 times with TBS-T, the membranes were probed with corresponding peroxidase conjugated secondary antibodies rabbit anti-mouse or goat anti-rabbit (Abliance former Paris, France) (1: 4000) at RT for 1 h. Detection was carried out by using a chemiluminescence detection kit (Clarity Western ECL substrate, Biorad). After washing steps, signals were detected with ChemiDoc MP (Biorad).

The relative intensity of bands was densitometrically determined by Image J software 1.46r (NIH, USA). For statistical analysis, all data from 3 independent experiments were expressed as the ratio to optical density values of the corresponding β-actin control [53]. The values were expressed as a percentage of the control group arbitrarily set at 100%. The statistical analysis was done with a Student t-test.

#### Fluorescence-activated cell sorting (FACS) by flow cytometry

After 24 h of treatment with 100 μM 5-HIAA, SH-SY5Y cells were harvested, centrifuged for 10 min at 1000 x g at RT and re-suspended with 0.5% PBS-BSA before being gently fixed in 4% paraformaldehyde (PFA, Sigma-Aldrich) in PBS for 1 h. Thereafter, the cells were submitted to two centrifugation steps (10 min at 1000 x g) separated by a washing step using 0.5% PBS-BSA. Cells were then incubated in permeabilization buffer (0.1% Triton X-100 in 0.1% sodium citrate) for 2 min on ice. An additional round of washing and centrifugation was performed before incubating the cells with anti-NEP antibody (Merck-Millipore; 1:200) in PBS for 25 min at 4 °C. At the end of the incubation, the cells were washed and centrifuged again for 10 min at 1000 x g. Supernatants were removed and the pellets were suspended and incubated with a secondary antibody for 25 min at 4 °C. The cells were washed and centrifuged a last time, the supernatants were removed, and the pellets were suspended in PBS before being analyzed.

#### Proteome profiler Phospho-MAPK array

After ½ hour of treatment with 100 μM 5-HIAA or without treatment, SH-SY5Y cells were harvested, centrifuged for 5 min at 300 x g at RT, and then solubilized at 1 × 10^7^ cells/ml in the Lysis Buffer provided with the kit for ½ hour. The level of phosphorylation was determined by using a Proteome Profiler Antibody Kit (Human Phospho-MAPK Array Kit, R&D Systems Europe, Oxford, UK). Briefly, two nitrocellulose membranes, containing 26 different capture antibodies printed in duplicate, were blocked for 1 h under agitation at RT. During the same time, the protein extracts were incubated with a detection antibody cocktail. After one hour, the blocking buffer was replaced by the mix protein extract - antibody cocktail and incubated overnight at 2–8 °C under agitation. Then the membranes were washed 3 times in wash buffer, and were put in a solution of Streptavidin-HRP for ½ hour under agitation at RT. After a further 3 washes, the membranes were covered with a Chemi-reagent mix for 1 min before detection of the signal with ChemiDoc MP (Biorad). The relative intensity of bands was densitometrically determined by Image J software 1.46r (NIH, USA).

#### Neprilysin activity assay

SH-SY5Y cells were harvested and washed with PBS after 0, 30, 60 and 90 min of treatment with 30 μM 5-HIAA (Sigma-Aldrich). The cells were then sonicated in iced Tris buffer (50 mM Tris-HCl, pH 7.4) and store at − 80 °C.

Measurement of neprilysin activity was performed according to the technical details given by the manufacturer for the fluorescent SensoLyte 520 kit (AnaSpec Inc., Fremont, CA, US). Briefly, 100 μg of total protein were placed in the wells of a non-binding 96-well plate (Corning) before adding the substrate working solution. The reagents were mixed by shaking the plate gently for 30 s and the fluorescence signal was immediately measured at Ex/Em = 490 nm/520 nm continuously and the data were recorded every 5 min for 1 h.

The initial reaction velocity was determined by the slope of the linear portion of the data plot and the results were expressed in percentage by reference to control condition.

#### Immunocytochemistry

Coverslips were prepared with SH-SY5Y cells treated or not by 5-HIAA at a concentration of 100 μM. The cells were fixed in 4% PFA for 15 min and then permeabilized with 100% ethanol for 5 min and incubated in PBS with 5% fetal bovine serum overnight at 4 °C. A rabbit polyclonal anti-neprilysin antibody (Merck; 1:200) was applied for 2 h at RT then a pre-adsorbed Goat polyclonal anti-Mouse IgG - H&L (Alexa Fluor® 647) antibody (Abcam; 1:500) was used as secondary antibody. Coverslips were mounted in glycerol mounting medium with DAPI and DABCO™ (Abcam) before microscopic analysis using a Zeiss microscope equipped for fluorescence.

### In vivo experiments

#### Animals and treatments

Procedures involving animals and their care were conducted in compliance with a European Communities Council Directive (86/609/EEC) and under the supervision of authorized investigators. In addition, all the protocols were reviewed and approved by the Alsace Head Office of the French Department of Veterinary and Public Health Guide for the Care and Use of Laboratory Animals. Mice were individually housed per cage in a room with 12/12-h light-dark cycle. The room was maintained under constant temperature and humidity conditions. Water and food were available ad libitum*.*

#### Mice models

Two mice models were used:

- Female Swiss albino mice, 3-month-old, outbreeded, about 20 g (Janvier Labs, France),
- Male APPSWE hemizygote mice (B6, SJL-Tg (APPSWE) 2576 Kha, tested for heterozygous RD1, Taconic Europe, Denmark) carrying a transgene coding for the 695-amino acid isoform of human Alzheimer APP (Tg 2576) and the corresponding wild type (WT), 13-month-old. All animals were 30-35 g body weight.

#### Phosphoramidon model of NEP inhibition and Aβ 1–40 or Aβ 1–42 quantification

Four groups of 5 Swiss Albino mice were treated for 5 consecutive days. Group 1 received the NEP inhibitor Phosphoramidon (Peptide Institute, Osaka, Japan) each day via intranasal route. Phosphoramidon was dissolved in PBS (with 1 mM ascorbic acid) at a concentration of 30 mM and was administered intranasally as previously described [28]. Group 2 was treated with 5-HIAA via intranasal route (24 μL of a 30 mM solution). Group 3 received PBS alone. Group 4 received both phosphoramidon and 5-HIAA.

### Aβ 1–40 and Aβ 1–42 quantification

After 5 days of treatment, the mice were euthanized for tissue collection 2 h after the last administration. The brains were removed, the two hemispheres separated and immediately frozen in liquid nitrogen and stored at − 80 °C.

The tissues of one hemisphere were homogenized in 8 volumes of ice-cold guanidine buffer (5.0 M guanidine-HCl/50 mM Tris-HCl, pH 8.0). Homogenates were mixed for 3–4 h at RT. The quantification of total proteins was done with a BCA protein assay kit (Pierce, Rockford, IL, USA). The lysates were then aliquoted and stored at − 80 °C until quantification of Aβ 1–40 and 1–42 peptides, according to the technical details given by the manufacturer (Fisher former Invitrogen, Carlsbad, CA, USA).

Samples were briefly diluted with cold Reaction buffer and centrifuged at 16,000 g for 20 min at 4 C. Supernatant was decanted and sample was stored on ice until use. Hundred μL of the diluted homogenates or of the Aβ peptide standards were incubated on the pre-coated 96-well plate for 2 h at RT. The plate was washed 4 times and 100 μL of the primary antibody was added for 1 h at RT. After 4 further washes, 100 μL of the secondary antibody was added for 30 min at RT. After 4 new washes, 100 μl of Stabilized Chromogen was added to each well for 30 min at RT in the dark. Finally, the reaction was stopped by 100 μL of Stop Solution to each well and the optical density was read at 450 nm in a plate reader (Elisa reader model Sigma 960, Metertech, Taipei, Taiwan).

The β peptides levels were interpolated from the standard curve generated from serial dilutions of synthetic peptide. Each measurement was repeated on 3 occasions, and the average value was calculated.

The results were analyzed with an analysis of variance (ANOVA *p* < 0.0001) to evaluate the effects of genotype and treatment factors. Post hoc analyses were performed with the Bonferroni test.

#### Tg 2576 and WT mice treatment

Two groups of mice (12 Tg 2576 and 12 WT) were administered daily via intra-nasal route with 4 μL of a 30 mM solution of 5-HIAA (Sigma-Aldrich, France) in PBS [20, 21]. In parallel, 24 other animals (12 Tg 2576 and 12 WT) were treated for 5 consecutive days with 48 mg/kg 5-Hydroxy-L-tryptophan (5-HTP, Sigma-Aldrich) administrated IP. Control groups were obtained by administration of PBS (12 Tg 2576 and 12 WT). These mice were the used for the neprilysin activity assay or for the spatial novelty task.

#### Neprilysin activity assay

To measure NEP activity in the mouse brain, the animals were treated daily for 5 consecutive days and euthanized for tissue collection 2 h after the last administration for the Tg 2576 mouse (sub-chronical treatment) and at various times (0 to 120 min) for the Swiss mice (acute treatment). The brains were removed, the two hemispheres separated and immediately frozen in liquid nitrogen and stored at − 80 °C. The tissues of one hemisphere were homogenized in 5 volumes of ice-cold PBS, centrifuged at 15000 x g for 5 min at 4 °C and the supernatant was decanted and stored at − 80 °C. The quantification of total proteins was done by a BCA protein assay kit).

Measurement of neprilysin activity was performed according to the technical details given by the manufacturer for the fluorescent SensoLyte 520 kit (AnaSpec Inc., Fremont, CA, US). Briefly, 100 μg of total protein were placed in the wells of a non-binding 96-well plate (Corning) before adding the substrate working solution. The reagents were mixed by shaking the plate gently for 30 s and the fluorescence signal was immediately measured at Ex/Em = 490 nm/520 nm continuously and the data were recorded every 5 min for 1 h.

The initial reaction velocity was determined by the slope of the linear portion of the data plot and the results were expressed in percentage by reference to control condition.

#### Spatial novelty task

This task is based on the spontaneous tendency of mice to preferentially explore objects which have been displaced within a familiar arrangement of objects. Tg 2576 mice are deeply impaired in this task as early as 7 to 8 months of age, independently of the rd mutation [38, 57].

The spatial novelty task was performed in a square Plexiglas open field (52 × 52 × 40 cm). For each testing period, a specific set of 7 different objects (4 for the habituation phases and 3 for the spatial recognition task), which differed in shape, color, and materials, were used. Each object was available in duplicate (one for each trial when required) and was wiped with 70% ethanol as the whole open field between each trial. On days 1 and 2, the mice received a habituation trial of 10 min with 2 different objects each day.

On day 3 (5th day of treatment), the mice explored the spatial configuration of 3 new objects during a 10-min acquisition trial, returned in their home cage for 3 min, and then received a 10-min retention trial with a new spatial configuration resulting from the shifting of one of the 3 objects to a new location. Recognition performances were calculated as the additional time spent exploring the displaced object during the retention trial when mice are the most involved in exploring the objects. This value was compared with the 0 value (no detection of the spatial change) with a Student t-test to measure whether spatial novelty detection occurred and was also compared among groups with an analysis of variance (ANOVA *p* < 0.0001) to evaluate the effects of genotype and treatment factors. Post-hoc analyses were performed with the Newman-Keuls test.

#### Immunocytochemistry on brain tissue slices

Tg 2576/APPSWE mice chronically treated with 5-HIAA were perfused through the heart with cold 4% PFA. The brains were fixed in a 4% PFA solution for 72 h at 4 °C and afterwards were immersed in PBS containing 20% sucrose for 48 h. The brains were cut in a vibratome Leica VT1000M (80 μm thick) and the slices were collected in a Watson medium.

Floating sections were immunostained as follows: Tissue slides were rehydrated with PBS for 1 h at RT then blocked overnight at 4 °C with 5% (*v*/v) fetal bovine serum in PBS. Sections were stirred overnight at 4 C with the primary antibodies (mouse monoclonal Anti-β Amyloid 1–40 antibody (Abcam) and rabbit polyclonal anti-Neprilysin antibody (Merck Millipore) at a dilution of 1:200. Sections were then washed 10 to 12 times for 1 h in PBS pH 7.4. Then the sections were stirred with species-specific secondary antibodies (pre-adsorbed Goat polyclonal anti-Mouse IgG - H&L (Alexa Fluor® 647) antibody (Abcam) and pre-adsorbed Goat polyclonal anti-Rabbit IgG - H&L (Alexa Fluor® 488) antibody (Abcam), at a dilution of 1:500, overnight at 4 C in the dark. Sections were washed again (10 to 12 times for 1 h) with PBS pH 7.4. After this second period of washing, the sections were mounted in glycerol mounting medium with DAPI and DABCO™ (Abcam) before microscopic analysis using a microscope (Zeiss AxioImager Z2).

### Statistical analysis

GraphPad Prism was used for all statistical analysis. For ANOVA multiple statistical comparisons, Newman-Keuls or Bonferroni tests were used and two-sided unpaired Student’s *t-*test for single statistical comparison. Statistical distribution for the Post-hoc analyses was: * *p* < 0.05, ** *p* < 0.005 and *** *p* < 0.0001. Errors are standard error of mean (SEM).

## Results

### The synthesis and activity of NEP in SH-SY5Y neuronal cells increase time-dependently in the presence of graded concentrations of 5-HIAA

**Fig. 1:**
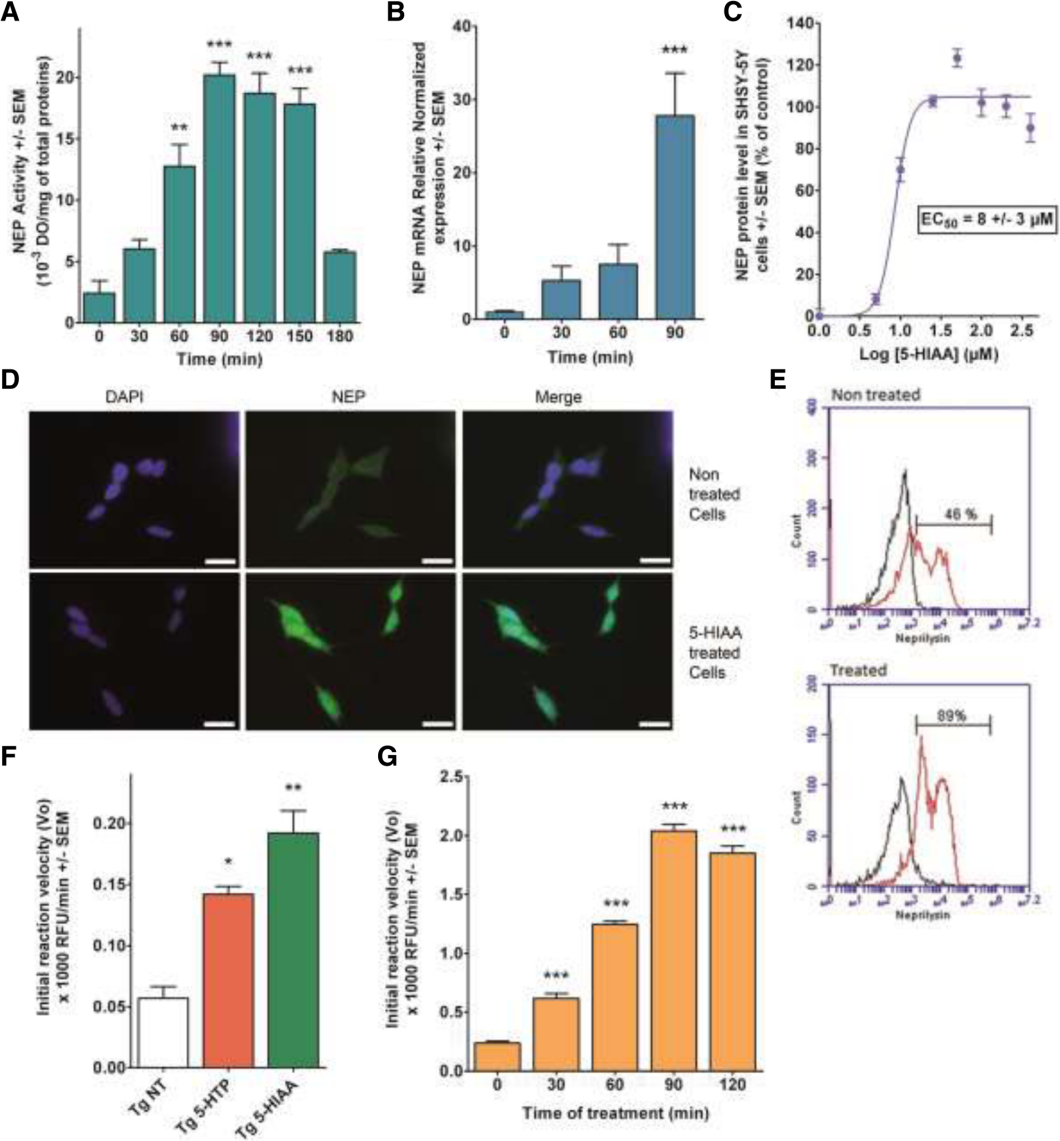
Effects of 5-HIAA on NEP expression and activity in SH-SY5Y neuronal cells and mouse brain. **a**, **b**, **c**: Experiments in SH-SY5Y neuroblastoma cells. **a** Kinetic of NEP activity as a function of incubation time (0 to 180 min) in cells treated by 100 μM 5-HIAA. NEP activity was determined with a fluorescent SensoLyte 520 kit (AnaSpec Inc., Fremont, CA, US). NEP activity culminates at 90–120 min after addition of the compound, with quantitative values corresponding to 20 +/− 1 and 19 +/ 2 10^3^ DO/mg of total proteins, respectively. These maximal values decrease to basal level after 3 h. **b** q-PCR assessment of NEP mRNA increase as a function of incubation time (0 to 90 min) in SH-SY5Y cells treated by 100 μM 5-HIAA. The increase of NEP mRNA level already detectable at 30 min, reached a value 30 times higher at 90 min which represents the time point corresponding to NEP maximal activity. **c** ELISA test determination of the dose-response effect of 24 h 5-HIAA treatment on NEP activity. Evaluation of the effects of increasing doses of 5-HIAA showed an EC_50_ of 8 ± 3 μM, a concentration within the range of 5-HIAA concentration found in brain tissue. The statistical analysis of triplicate samples was done by an ANOVA completed with a Bonferroni’s Multiple Comparison Test. ** *p* < 0.005, *** *p* < 0.0001. **d** Immunocytochemistry with anti-NEP antibody of SH-SY5Y cells treated with 100 μM 5-HIAA for 24 h versus non-treated controls. Scale bars: 20 μm. **e** Fluorescence-activated cell sorting (FACS) by flow cytometry on SHSY-5Y cells treated with 5-HIAA (100 μM) for 24 h versus controls. 46% of cells were neprilysin-positive in control cells while 89% were positively labeled in treated cells. Black curve: no secondary antibody; Red curve: cells treated with NEP secondary antibody. **f**, **g**: Experiments on mouse brain in vivo. **f** Effects of 5-HTP (48 mg/kg, I.P.) or 5-HIAA (24 μL of a 30 mM solution, intra-nasal) on NEP activity in 14 months APPSWE mice brain (*n* = 5 for each treatment). Drugs were administered each day during five consecutive days. Both 5-HTP and 5-HIAA treatments increase the level of NEP activity in brain (142 +/− 7 and 192 +/− 18 Relative Fluorescence Units (RFU)/min, respectively). However, 5-HIAA treatment was the most effective. **g** Kinetics of NEP enzymatic activity in 3 months Swiss Albinos mice (*n* = 5 at each time point) treated by an intra-nasal administration of 24 μL of a 30 mM solution of 5-HIAA at time zero. The brain NEP activity was determined every 30 min until 120 min. A rapid increase of NEP activity was detected at 90 min with a maximum value of 2040 +/− 50 RFU/min. The statistical analysis was done by an ANOVA completed with a Bonferroni’s Multiple Comparison Test. * *p* < 0.05; ** *p* < 0.005; *** *p* < 0.0001 by reference to NT or time zero

The serotonin catabolite 5-HIAA at a dose of 100 μM increases NEP activity in SH-SY5Y cells with time. The maximum activity culminates at 90–120 min after addition of the compound and decrease to basal level 3 h later (Fig. 1a). Under the same conditions, the corresponding NEP mRNA messenger was measured in cell cultures until 90 min of incubation. An increase was noticed starting at 30 min and was elevated by about 30 times after 90 min, which corresponds to the maximum of NEP activity (Fig. 1b). The neo-synthesis of NEP protein, measured in cells cultured in the presence of increased amounts of 5-HIAA by an ELISA method, showed an EC_50_ of 8 ± 3 μM (Fig. 1c). This concentration is within the range of 5-HIAA concentration found in brain tissue [50].

### 5-HIAA or 5-HTP administration in vivo increases NEP activity in the mouse brain

Tg 2576 mice were intranasally administered either 24 μL of a 30 mM solution of 5-HIAA or with 48 mg/kg I.P of 5-HTP consecutively for 5 days. At the end of treatment, NEP activities were measured. Both 5-HTP and 5-HIAA treatments increase the level of NEP activity in brain, the treatment with 5-HIAA being the more active (Fig. 1f). In a second series of experiments, Swiss albino mice were treated with 5-HIAA at time zero (24 μL of a 30 mM solution) and the NEP activity was determined in brain every 30 min until 120 min. A rapid increase in this activity was shown with a maximum at 90 min, confirming in vivo the result obtained in cell cultures (Fig. 1g).

### Cell analysis by flow cytometry confirms the stimulatory effect of 5-HIAA on NEP expression

The induction of NEP expression by 5-HIAA is rapid when measured in a cell’s homogenate. However, NEP protein targeting the cell membrane is a prolonged process [25]. For this reason, we confirmed the induction of NEP protein in SH-SY5Y neuronal cells using flow cytometry analysis after incubation for 24 h with 100 μM 5-HIAA. The cells were labeled with a NEP specific antibody. Figure 1d represents a qualitative analysis of the intensity of NEP antibody-induced immunolabeling in 5-HIAA-treated and control SH-SY5Y cells. A quantitative approach has been used to count cells by flow cytometry. Figure 1e shows the percentage of NEP positive cells under control conditions (46%) and in the presence of 100 μM 5-HIAA (89%).

### Effect of 5-HIAA in a pharmacological model of brain Aβ accumulation

**Fig. 2:**
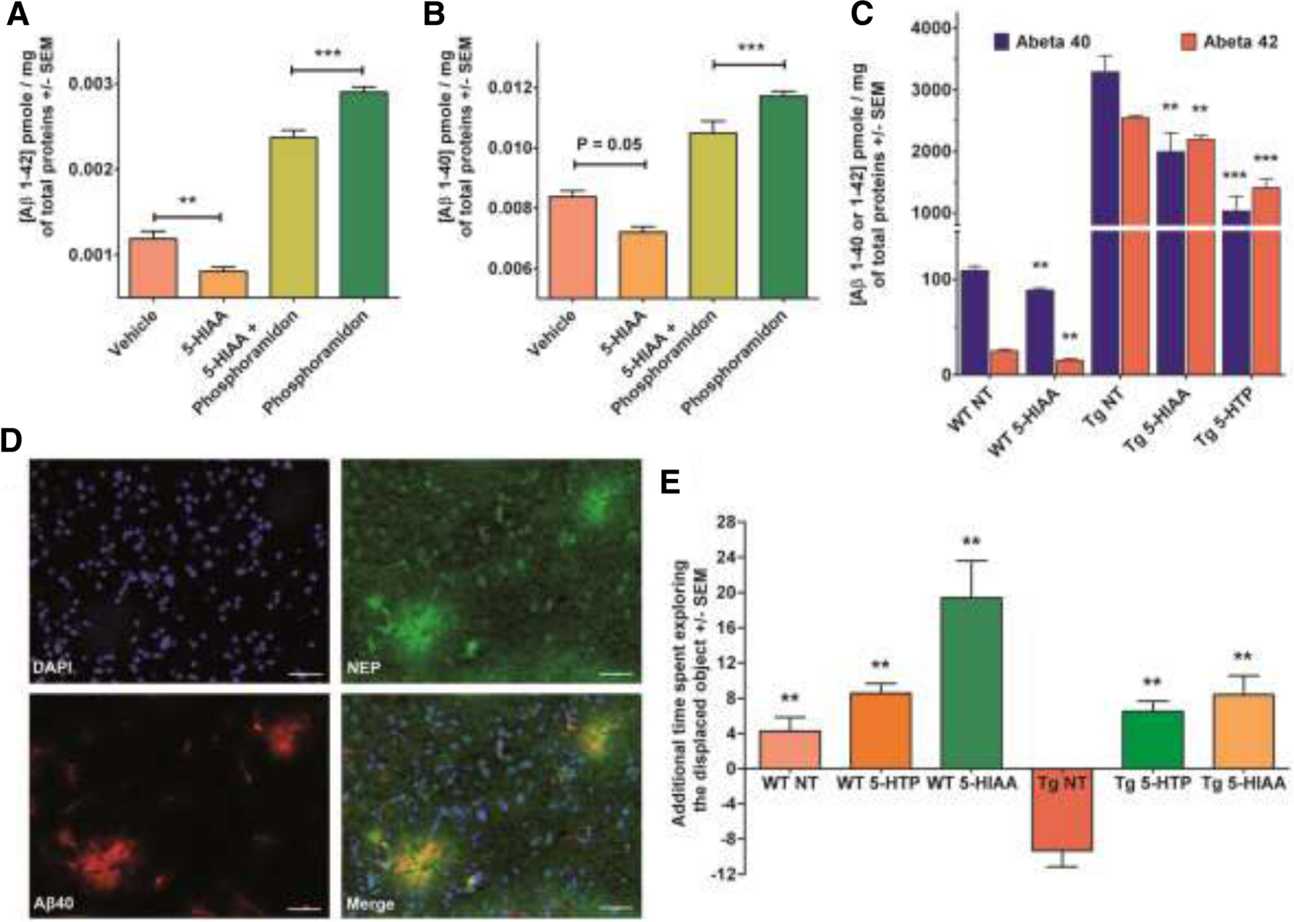
Effects of 5-HIAA on brain amyloid peptide accumulation and memory performance. **a**, **b**: Inhibitory action of 5-HIAA against phosphoramidon-induced brain Aβ accumulation in 3-month-old Swiss albino mice (*n* = 7 for each group). **a** Phosphoramidon induces an increase in Aβ 1–42 peptide which is counteracted by 5-HIAA treatment. The effect of 5-HIAA can be seen also in control mice non-treated with Phosphoramidon (see also Table 1). Statistical analysis by an ANOVA completed with a Bonferroni’s Multiple Comparison Test. * *p* < 0.05, ** *p* < 0.005, *** *p* < 0.0001. **b**: Same results than in A with the Aβ 1–40 peptide. **c** 5-HIAA- or 5-HTP-induced decrease of brain Aβ peptide level in 14-month-old Tg 2576/APPSWE mice (Tg). The mice (*n* = 7 for each group) were treated daily during 5 consecutive days with 5-HTP (48 mg/kg, I.P.) or 5-HIAA (24 μL of a 30 mM, intra-nasal). 5-HIAA treatment reduces brain 1–40 and 1–42 Aβ concentrations in wild type (WT) and APPSWE mice. 5-HTP treatment is also active in APPSWE mice (see also Table 2). The statistical analysis was done by an ANOVA completed with a Bonferroni’s Multiple Comparison Test of NT (non-treated) WT (wild-type) or Tg (Tg 2576/APPSWE) mice. **d** Photomicrographs showing the co-localization of NEP and 1–40 Aβ peptide in the cortex of Tg 2576/APPSWE mice brain. Scale bars: 50 μm. This image is an illustration of the distribution of NEP and amyloid deposits co-localization. **e** Effects of 5 consecutive days intra-nasal 5-HIAA (24 μl of a 30 mM solution) or I.P. 5-HTP (48 mg/kg) treatments on the spatial novelty performances of 14-month-old wild-type (WT) and Tg 2576/APPSWE (Tg) mice (*n* = 7 for each group). Results are shown as the mean additional time (SEM) spent in exploring the displaced object versus the non-displaced objects during the retention trial. This value was compared to the 0 value (no detection of the spatial change) with a Student t-test (** *p* < 0.01) to determine whether spatial novelty detection occurred. Treated WT mice exhibited an improved performance compared to control animals; 5-HIAA-treated mice showed the best performance. Non-treated (NT) Tg 2576/APPSWE mice performed very poorly compared to treated transgenic animals. The various groups were compared with an ANOVA (*P* < 0.0001) completed with a Newman-Keuls Multiple Comparison Test to evaluate the effects of treatments. Retention performances of the non-treated (NT) APPSWE groups were lower than those of treated APPSWE, treated WT and non-treated WT groups, respectively

Adult Swiss albino mice treated for 5 days by intranasal administrations of the NEP inhibitor phosphoramidon (K_i_ of about 2 nM) showed increased brain concentrations of both 1–40 Aβ (+ 39%) and 1–42 Aβ (+ 144%) compared to control mice treated with saline. Administration of 5-HIAA alone to healthy mice reduced brain basal levels of 1–40 and 1–42 Aβ about 14 and 32%, respectively. The co-treatment of phosphoramidon-receiving mice with 5-HIAA decreased the brain 1–40 Aβ (− 15%) and 1–42 Aβ (− 45%) concentrations. These results reveal that 5-HIAA is capable of counteracting Aβ accumulation in the brain under physiological or pharmacologically-evoked (phosphoramidon-induced NEP inhibition) conditions (Fig. 2a and b and Table 1).

**Table 1:** Variations of brain amyloid peptide level in phosphoramidon-treated and control mice after 5-HIAA or vehicle treatment

	Vehicle	5-HIAA	Phosphoramidon	5-HIAA + Phosphoramidon	
Aβ42	1.2	0.8	2.9	2.4	Mean (pg/total proteins)
	100	68	244	199	Mean compared with vehicle (%)
	0	−32	144	99	% of variation compared to Vehicle
	−144	− 176	0	−45	% of variation compared to Phosphoramidon
Aβ40	8.4	7.2	11.7	10.5	Mean (pg/total proteins)
	100	86	140	125	Mean compared with vehicle (%)
	0	−14	40	25	% of variation compared to Vehicle
	−39	−54	0	−15	% of variation compared to phosphoramidon

### 5-HIAA treatment reduces brain Aβ concentration in APPSWE mice

**Fig. 3:**
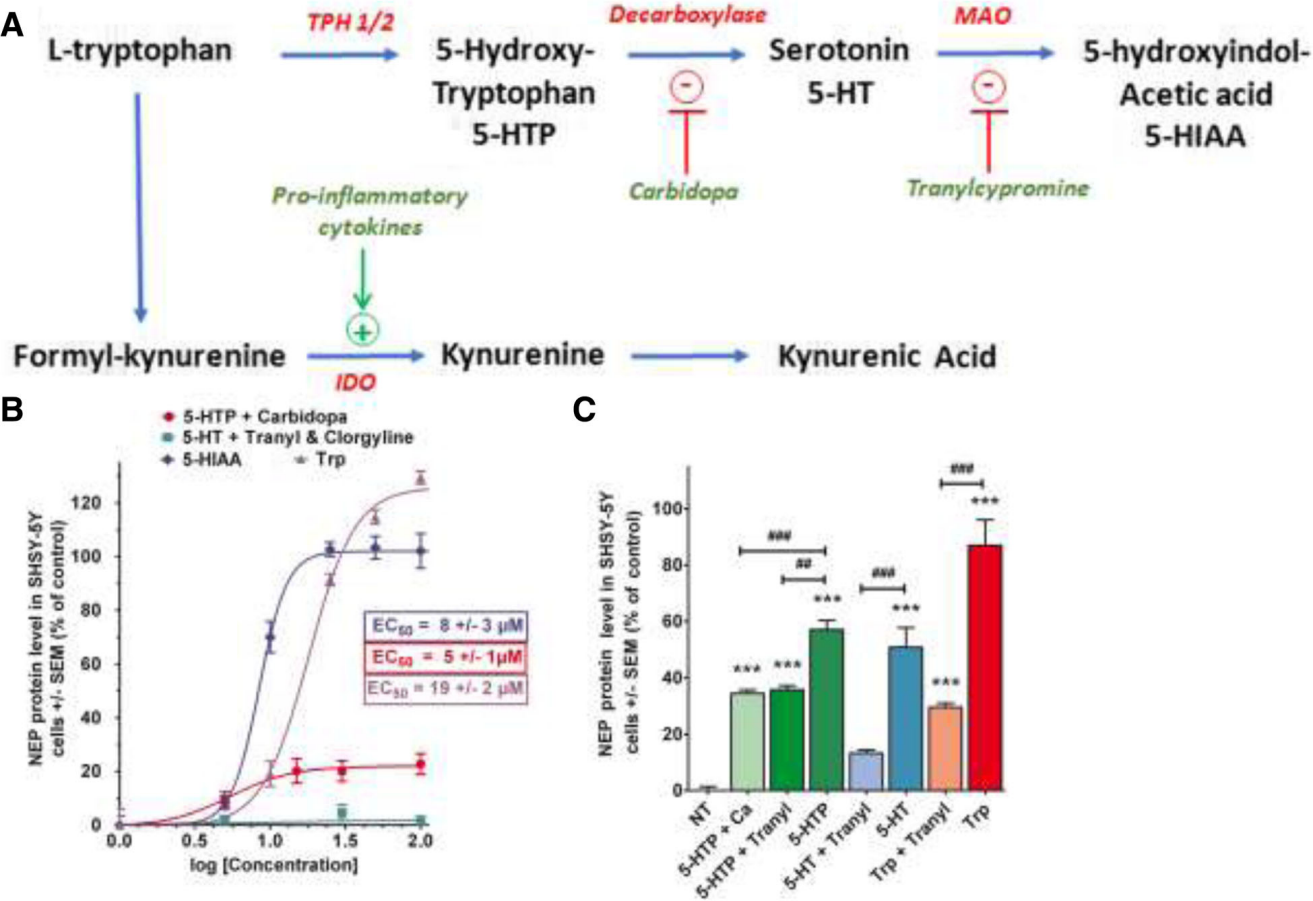
Role of 5-HIAA precursors on NEP protein expression. **a** Tryptophan is the precursor of both serotonin and kynurenine pathways. IDO (indolamine dioxygenase 2,3 dioxygenase) induction by inflammatory cytokines favorizes the production of kynurenine pathway intermediates, including kynurenic acid. Tryptophan, 5-hydroxytryptophan (5-HTP) and serotonin (5-HT) are precursors of 5-HIAA. 5-HTP degradation is prevented by carbidopa while serotonin catabolism is blocked by IMAO (tranylcypromine + clorgyline). **b** Dose-response curves for the induction of NEP protein in SH-SY5Y cells. Effect of tryptophan, 5-HIAA, 5HTP + carbidopa (100 μM), Serotonin + tranylcypromine (30 μM) and clorgyline (20 μM) on NEP protein level in SH-SY5Y cells after 30-min treatment. In the absence of inhibitor, 5-HIAA and L-tryptophan exhibit an EC_50_ of 8 ± 3 μM and 19 ± 2 μM for NEP induction, respectively. In the presence of carbidopa, 5-HTP shows an intrinsic activity on NEP induction with an EC_50_ of 5 ± 1 μM but the maximum activity is 20%. Incubation of SH-SY5Y cells with increasing doses of serotonin in the presence of tranylcypromine showed no effect on NEP induction. **c** Effects of 24-h pharmacological inhibition of 5-HIAA synthesis from precursors (Tryptophan, 5-HTP, or 5-HT) on NEP protein expression in SH-SY5Y cells. In the presence of Tranylcypromine, NEP induction by tryptophan is reduced from 87 +/− 9 to 30 +/− 1%. In the presence of either carbidopa (inhibitor of 5-HTP decarboxylation into 5-HT), or tranylcypromine (blocker of 5-HTP conversion into 5-HIAA), NEP induction by 5-HTP was significantly reduced compared to 5-HTP tested without inhibitors (respectively 35 +/− 1 and 36 +/− 1% vs 57 +/− 3%). 5-HT at 100 μM exhibited some effect on NEP (51 +/− 7%) because of its role as a precursor of 5-HIAA. Consistently, the effect of 5-HT was inhibited by tranylcypromine (13 +/− 1%). The statistical analysis of triplicatesamples was done by an ANOVA completed with a Bonferroni’s Multiple Comparison Test. ^##^ *p* < 0.005, ^###^ or *** *p* < 0.0001. * by reference to non-treated (NT) cells, or # by reference to treatments with specific enzyme’s inhibitor

Tg 2576 APPSWE mice are well characterized as a model for AD-related Aβ pathology expressing brain 1–40 and 1–42 Aβ accumulations [17, 22, 54]. We measured the concentration of 1–40 and 1–42 Aβ peptides in cerebral hemispheres of 14 months aged APPSWE mice treated (5 consecutive days) or not by intranasal 5-HIAA or by 5-HTP (48 mg/kg) injected IP. This last compound is a precursor of 5-HIAA in the brain, but also of serotonin (Fig. 3a). Six hours after the last administration of each compound, the animals were killed and brain Aβ concentrations were determined.

In the wild type counterparts of Tg 2576 mice, 5-HIAA treatment reduces brain 1–40 and 1–42 Aβ concentrations by 19 and 39%, respectively. In APPSWE mice, the decrease of brain 1–40 Aβ level was − 39% and − 69% after 5-HIAA or 5-HTP administration while the reduction of cerebral 1–42 Aβ level was − 14% and − 45% after 5-HIAA or 5-HTP treatment, respectively (Fig. 2c and Table 2).

**Table 2:** Variations of brain amyloid peptide level in APPSWE and WT mice after 5-HIAA or 5-HTP treatment

	WT NT	WT 5HIAA	Tg NT	Tg 5HIAA	Tg 5HTP	
Aβ40	109.2	88.6	3285	1996	1031	Mean (pg/total proteins)
	100	81	100	61	31	Mean compared with NT (%)
	0	−19	0	−39	−69	% of variation compared to corresponding NT
Aβ42	25.2	15.3	2544	2192	1408	Mean (pg/total proteins)
	100	61	100	86	55	Mean compared with NT (%)
	0	−39	0	−14	−45	% of variation compared to corresponding NT

### 5-HIAA or 5-HTP treatments ameliorate memory task performance in both WT and Tg 2576 APPSWE mice

Tg 2576 APPSWE mice and their WT counterparts were treated for five consecutive days with 5-HIAA (intranasal administration) or by 5-HTP (48 mg/kg I.P). Six hours after the last administration, the mice were tested for spatial recognition test. WT mice treated with 5-HTP or 5-HIAA exhibited an improved performance than control animals treated with saline; 5-HIAA-treated animals showed the best performance. Non-treated Tg 2576 APPSWE mice performed very poorly compared to transgenic animals treated with 5-HTP or 5-HIAA under the same conditions. Retention performances of the non-treated APPSWE groups were lower than those of treated APPSWE, treated WT and non-treated WT groups respectively (Newman-Keuls: *p* < 0.001, APPSWE control groups compared to each other group) (Fig. 2e).

### A role for 5-HIAA precursors in NEP induction?

Tryptophan, 5-HTP and serotonin (5-HT) are the main precursors of 5-HIAA in the brain. We examined in a series of experiments the potential role of these compounds to induce NEP via the production of 5-HIAA. Tryptophan is both the precursor of kynurenic acid through the kynurenic pathway and of 5-HIAA through serotonin catabolism (Fig. 3a). Kynurenic acid has been found to be a NEP inducer and its neuroprotective properties are well documented [29]. In the presence of IMAO (A + B), the induction of NEP by tryptophan is reduced by about 50%, so the remaining activity of tryptophan could be due to the formation of kynurenic acid. In the absence of inhibitor, L-tryptophan possesses an EC_50_ of 19 ± 2 μM for the induction of NEP in SH-SY5Y cell cultures (Fig. 3b and c).

5-HTP is a well-known precursor of serotonin and of 5-HIAA in the brain. In the presence of either carbidopa, which inhibits its decarboxylation into serotonin or of tranylcypromine which blocks its transformation into 5-HIAA, the inductive capacity of 5-HTP on NEP expression is significantly reduced compared to 5-HTP tested without inhibitors (Fig. 3c). In the presence of carbidopa, 5-HTP possesses an intrinsic activity on NEP induction with an EC_50_ of 5 ± 1 μM (Fig. 3b), but the maximum activity reaches a plateau at 20%.

Finally, increasing doses of serotonin incubated in the presence of tranylcypromine showed no effect on NEP induction (Fig. 3b, green). Also, serotonin at 100 μM exerted no significant effect on NEP expression in the presence of IMAO (Fig. 3c). Serotonin incubated alone exhibited some effect, probably because of its role as a precursor of 5-HIAA.

### 5-HIAA induces NEP neo-expression via a MAP-kinase driven mechanism

**Fig. 4:**
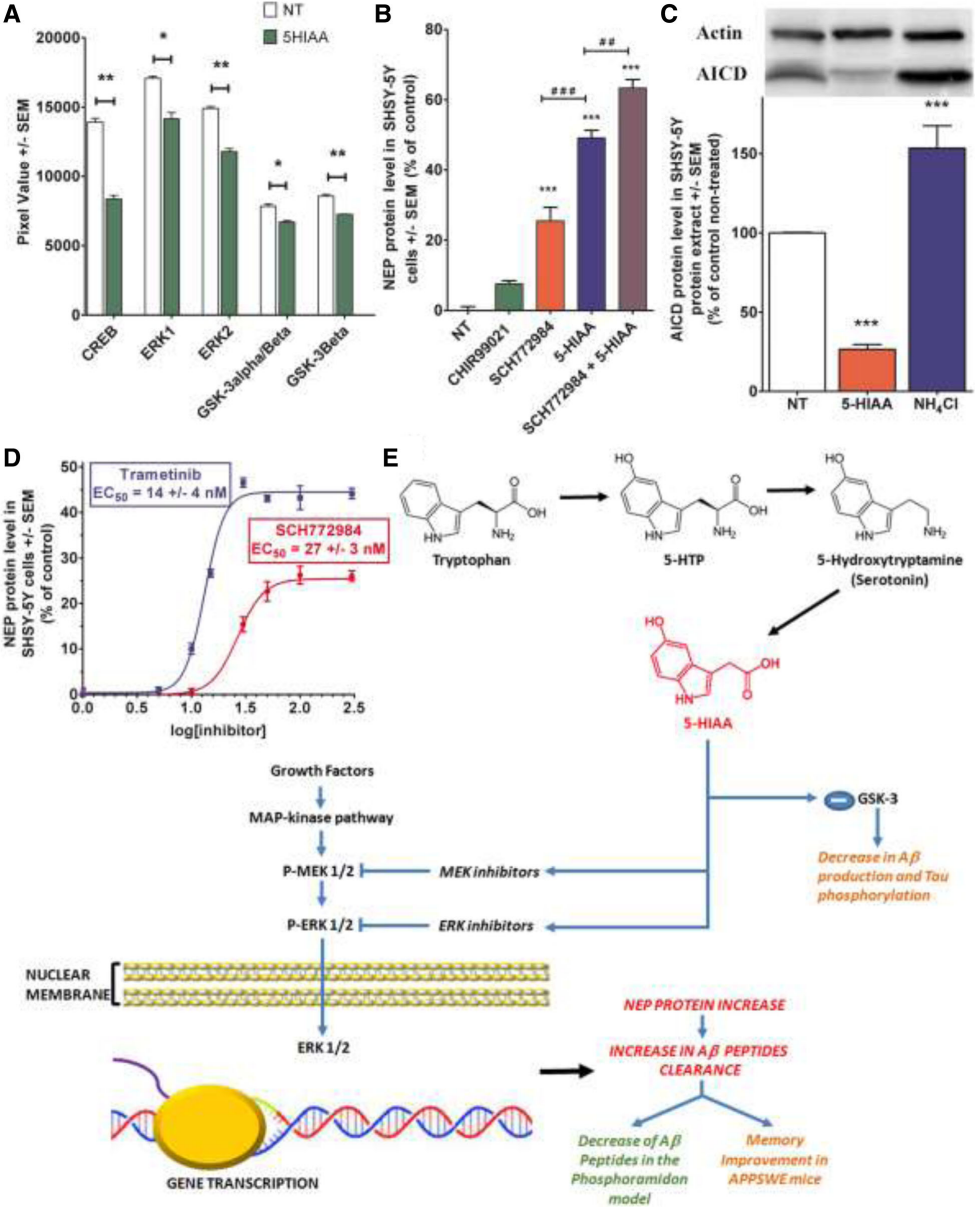
5-HIAA induces NEP expression via the MAP-kinase/ERK ½ pathway. **a** Down-regulatory effect of 5-HIAA on ERK ½, MEK ½ and GSK-3 expression in SH-SY5Y cells after 30 min treatment. The ERK pathway was down-regulated by 5-HIAA (mean decrease of ERK1–17% and ERK2–21%). GSK-3 α/β, a strategic factor for tau phosphorylation, was also significantly affected by the treatment (mean decrease of GSK-3α/β and GSK-3β − 15%), together with the transcription factor CREB (mean decrease − 40%). The statistical analysis of triplicate samples was done with a Student t-test. * *p* < 0.05, ** *p* < 0.005 by reference to non-treated cells (NT). **b** Effects of separated or combined treatments with the ERK ½ inhibitor SCH772984 and/or 5-HIAA on NEP expression in SH-SY5Y cells after 90 min treatment. NEP protein expression was stimulated by SCH772984 (26 +/− 4%) or 100 μM 5-HIAA (49 +/− 2%) tested separately. The combined treatment containing 100 μM 5-HIAA and SCH772984 showed a higher increase percentage of NEP expression (63 +/− 2%) compared to the separate treatments. The statistical analysis of triplicate samples was done by an ANOVA completed with a Bonferroni’s Multiple Comparison Test. ^##^ *p* < 0.005, ^###^ or *** *p* < 0.0001. * by reference to non-treated cells (NT), or ^#^ by reference to treatments with 5-HIAA. **c** Inhibitory action of 5-HIAA on AICD expression in SH-SY5Y cells. NH_4_Cl as positive control. The statistical analysis (*n* = 5 in each group) was performed by an ANOVA completed with a Bonferroni’s Multiple Comparison Test. *** *p* < 0.0001 by reference to non-treated cells (NT). **d** Dose-response effect and EC_50_ of SCH772984 and Trametinib on NEP expression in SH-SY5Y cells. Both MEK inhibitor (GSK 1120212, Trametinib) and ERK kinase inhibitor (SCH772984) induce NEP with an EC_50_ of 14 +/− 4 and 27 +/− 3 nM, respectively. **e** Graphical summary of potential mechanisms involved in 5-HIAA-induced NEP expression. The MAP-kinase pathway is involved in NEP expression in SH-SY5Y cells: inhibition of MEK/ERK ½ phosphorylation increased NEP protein levels. Phosphorylation of GSK-3 is also down-regulated by 5-HIAA treatment. These effects may explain 5-HIAA-induced increase in Aβ peptide clearance

The level of phosphorylation of the principal intermediates of MAP-kinase pathway was determined by using the Proteome Profiler Antibody Kit (R&D Systems). This system detects the phosphorylation of 26 human kinases simultaneously. SH-SY5Y cells were treated during 30 min with 100 μM 5-HIAA and then were analyzed according to the recommended protocol. Figure 4a represents the integrated spots that were significantly different from the non-treated cells. From these results, we decided to explore particularly the ERK pathway, which appears to be down-regulated by 5-HIAA. The modification of GSK-3 α/β, a strategic factor for tau phosphorylation, was also significantly affected by the treatment, together with the transcription factor CREB. The phosphorylation of all these intermediates and transcription factor are decreased by the cell’s treatment with 5-HIAA.

SCH772984 is a specific inhibitor of ERK1/2 with IC_50_ values of 4 nM and 1 nM in cell-free assays [8]. We incubated this compound alone or in combination with 5-HIAA in SH-SY5Y incubation medium to confirm the role of ERK pathway inhibition in the neo-expression of NEP. As shown in Fig. 4b, NEP protein expression is strongly stimulated by the presence of SCH772984 after 90 min of incubation and the addition of 100 μM 5-HIAA increases this stimulation significantly. Similarly, the MEK inhibitor (GSK 1120212, Trametinib) which plays a role as a MEK/MAPK/ERK kinase inhibitor, induces NEP with an EC_50_ in the nanomolar range. These results constitute additional evidence for a role of MAP-kinase cascade in NEP expression mediated by 5-HIAA. However, as several reports described a role for AICD peptide (intra-cellular domain of APP) in the control of NEP expression [18], this possibility was tested in the same experimental model. While NH_4_Cl strongly induced AICD, this peptide was down-regulated by 5-HIAA, suggesting that the mechanism activated by 5-HIAA to induce NEP expression does not involve or depend on AICD stimulation (Fig. 4c).

## Discussion and conclusions

In its early stage of clinical expression, sporadic AD is heterogeneous with cognitive, behavioral or psychological symptoms of many kinds, including alterations of mood, emotive disorders, confusion, agitation and anxiety or modifications in the sleep-wake cycle [46]. Serotonin and its brain innervations are known to modulate these alterations and pharmacological manipulations of the serotonergic system have long been used as therapeutic approach for these symptoms [11]. This therapeutic strategy includes the potentiation of serotonin synthesis by tryptophan or 5-HTP administration, the inhibition of serotonin transport by SSRI’s or degradation by IMAO. Among the large family of serotonergic receptors (5-HT1 to 5-HT7, each of them containing several isoforms), several classes appear to be implicated in AD related mechanisms. 5-HT2 receptors (2A, 2B, 2C) which activate phospholipase C pathway and 5-HT4, 5-HT6 that are coupled to Gs, have been reported to regulate the proteolytic cleavage of APP, neuroinflammation and cognitive deficits. The mechanisms involved in these processes are unknown, and serotonin is supposed to activate the complete set of its receptors with a global effect to be defined regarding AD [24]. This disease is characterized by diffuse brain atrophy due to neuronal degeneration and synaptic loss affecting multiple regions in the brain. The neurodegeneration results from Aβ accumulation and hyperphosphorylation of Tau which affect neuron survival [9]. Interestingly, it has been shown that the dorsal raphe nucleus exhibits cytoskeletal lesions at early stages of the disease, suggesting a possible role for the ascending serotonergic pathway in early manifestations of AD [5, 19, 43]. Amyloid protein accumulation in the brain is a prolonged process induced by chronic inflammation, reduction of energy metabolism and promotion of apoptotic mechanisms which precede the development of microtubule-associated protein Tau hyperphosphorylation and loss of functions [13]. Several mutations and gene variants are in favor of a closed link between Aβ proteinopathy and progressive occurrence of dementia in AD [1, 42]. The accumulation of amyloid oligomers is tightly regulated in the normal brain, in part by a fine control of their clearance. The cellular proteolysis of many brain proteins involves the proteasome and the lysosome, but also many proteolytic activities including Insulin-degrading enzyme, Endothelin or angiotensin converting enzyme and matrix metalloproteinases among others [33, 56]. However, a pivotal role is assigned to NEP, a cell-surface ectoenzyme degrading several key neuropeptides (enkephalins, substance P), which has been identified as a major Aβ degrading enzyme in the brain. Over-expression of NEP in Drosophila or in transgenic mice could suppress amyloid plaques formation or reduce brain Aβ levels. By contrast, a reduction of NEP expression has been evidenced in the brain of aged or AD patients [23, 33]. However, NEP activity seems to be stimulated in brain cells in contact with amyloid deposits in human brain. It appears that NEP up-regulation could be an adaptive mechanism counteracting brain Aβ accumulation and protecting thus neuron survival during the disease incubation [40]. Since several years, a pharmacological approach has been suggested to increase NEP activity in the brain and several compounds were identified as NEP expression modulators, some of them being potential actors regulating NEP homeostasis in vivo [28, 29, 34, 51]. The fundamental interest of these studies is to try to slow-down the occurrence of AD clinical symptoms and beneficial effects against memory deficits have been reported in animal models following NEP up-regulation.

The present work describes the role of 5-HIAA, the final product of serotonin metabolism, as an inducer of NEP expression in the brain. This compound is not a ligand for serotonin receptors and was until now considered only as a dead-end product, with no functional role. However, 5-HIAA was implicated recently in the negative control of RAS/MAPK signaling pathway in *C. elegans* [45]. Interestingly, NT-3 treatment significantly increased Aβ in primary neuron cultures, an effect which was abolished by the MEK 1/2 inhibitor UO126 [25]. The Ras/MAP-kinases pathway was reported to be elevated in initial stages of AD, before the accumulation and pathology driven by Aβ [15, 16]. A pathological link between Aβ generation and altered MAP/phospho-ERK signaling seems to exist. Phospho-ERK contributes to tau phosphorylation but GSK-3 is also involved in this process, together with several other kinases [27]. GSK-3 promotes every major pathological process in AD, including apoptosis and GSK-3 inhibitors improve several cognitive functions in transgenic mice models for AD [4]. We demonstrate that in SH-SY5Y cell cultures, 5-HIAA-induced neo-expression of NEP protein and this effect is partially reproduced by the inhibition of ERK 1/2 pathway. 5-HIAA reduces also the expression of GSK-3 and this action may additionally ameliorate AD symptoms in APPSWE transgenic mice. Moreover, P-CREB decrease observed after 5-HIAA administration may probably result from the down-regulation of MAP-kinase/GSK-3 pathways but it is difficult to estimate the direct impact of this effect on the behavior of APPSWE mice owing to the involvement of P-CREB in various events related to brain plasticity [7].

Independently from the effect of 5-HIAA on NEP gene expression, its possible inhibitory action at the hamster prion promoter controlling the mutated human APP isoform expression in APPSWE mice is worthy of attention. 5-HIAA may act via this mechanism to reduce mutated APP expression and decrease Aβ accumulation in APPSWE mice. However, the probability of such action appears extremely low since the removal of PrP^c^ prion from human APP-transgenic mice did not modify APP proteolysis, Aβ levels or pathologic phenotype [55].

Consistently, we observed that 5-HIAA increased NEP expression and decreased brain Aβ in the mouse model of intranasal phosphoramidon-induced NEP inhibition and cerebral accumulation of amyloid oligomers [21]. However, besides NEP inhibition, phosphoramidon may also reduce the activity of other Aβ degrading enzymes including the endothelin-converting enzyme [14]. Therefore, the possibility that in addition to NEP induction, 5-HIAA may also promote the activity of other Aβ degrading enzymes cannot be ruled out. Moreover, it has been reported that intracerebroventricular injection of the protease inhibitor phosphoramidon into wild type mice increased brain Aβ deposits and concomitantly induced neurodegeneration of hippocampal neurons and neuroinflammation [37]. Interestingly, we found that in the transgenic mouse model of AD expressing a human-mutated APP (APP Swedish) and accumulating brain Aβ, in vivo administration of 5-HIAA treatment during 5 consecutive days significantly reduced brain Aβ concentration and counteracted memory deficits in a spatial recognition task. The possibility exists that a longer period of treatment could induce a deeper elimination of Aβ, since there is no obvious toxicity of 5-HIAA. However, as the intra-nasal route was used to circumvent the blood brain barrier, this mode of administration may have some limitations for very long-term therapies. Therefore, a possibility could be the use of precursors of 5-HIAA such as tryptophan or 5-HTP that can be absorbed per os. Serotonin is also degraded into 5-HIAA in vitro and the global activity of the serotonergic system influences 5-HIAA levels in vivo. Thus, we explored a possible effect of these three substances using pharmacological treatment of SH-SY5Y neuronal cultures. We found that tryptophan, 5-HTP and serotonin induce NEP and these effects were strongly reduced by the presence of carbidopa or pargyline which inhibits dopa-decarboxylase or MAO, respectively. Thus, these three substances appear to act on NEP protein levels via the synthesis of 5-HIAA. For tryptophan, whose dietary increase reduces intraneuronal Aβ density in transgenic animals [32], the blockade of its degradation into 5-HIAA by MAO inhibition strongly reduced its ability to induce NEP synthesis. But tryptophan is the precursor of both kynurenine and serotonin pathways, and chronic inflammation is thought to drive tryptophan metabolism into the kynurenine pathway after induction of indolamine 2,3-dioxygenase, in detriment of serotonin metabolism [30]. A role of some intermediates of kynurenine pathway has been described in the pathophysiology of neurodegenerative diseases, including AD [6, 31]. Concerning 5-HTP, a direct precursor of both serotonin and 5-HIAA, the inhibition of dopa-decarboxylase decreases its effect on NEP induction, but this compound has also a direct role on NEP protein levels in cell cultures with an EC_50_ of about 5 μM.

The role of serotonin appears more complex. The synthesis of serotonin is associated with lower Aβ levels in animals and humans [10]. In the presence of a MAO inhibitor, the stimulatory effect of serotonin on NEP protein in cell cultures disappeared, indicating that the reported beneficial effect of serotonin on Aβ levels is partly due to its catabolism into 5-HIAA. Serotonin itself has no effect on NEP but this neurotransmitter has been reported to exert several beneficial effects on AD via other mechanisms, as demonstrated by the pharmacological action of some SSRI and agonists/antagonists at 5-HT receptors [47]. Moreover, it is well demonstrated that the raphe nuclei are affected by neurofibrillary tangles very early in AD pathogenesis, suggesting the occurrence of early serotoninergic alterations that may facilitate AD progression [5, 19, 43]. Therefore, our results showing a beneficial role and mechanisms of action of serotonin metabolite 5-HIAA (until now considered only as a dead-end inactive product) in a mouse model of AD, may contribute to improve the efficacy of serotoninergic derivatives-based strategies against AD.

In conclusion, the present report, which provides the first demonstration that 5-HIAA sub-chronic therapy actively increases brain Aβ degradation/clearance and ameliorates symptoms in the APPSWE mouse model for AD, also contributes to elucidate the role of tryptophan/kynurenine/serotonin interrelated pathways in AD pathophysiology and progression.

## Abbreviations

5-HIAA: 5-hydroxyindolacetic acid
APPSWE: Transgenic mice, B6
ERK ½: Extracellular signal-regulated kinases
GSK-3: Glycogen synthase kinase-3
MEK ½: Mitogen extracellular signal-regulated kinases
NEP: Neprilysin
SJL-Tg: (APPSWE) 2576Kha

## References

[1] Bagyinszky E, Youn YC, An SS, Kim S (2014). The genetics of Alzheimer's disease. Clin Interv Aging.

[2] Baranello RJ, Bharani KL, al PV (2015). Amyloid-beta protein clearance and degradation (ABCD) pathways and their role in Alzheimer's disease. Curr Alzheimer Res.

[3] Benosman S, Gross I, N C (2007). Multiple neurotoxic stresses converge on MDMX proteolysis to cause neuronal apoptosis. Cell Death Differ.

[4] Beurel E, Grieco SF, Jope RS (2015). Glycogen synthase kinase-3 (GSK3): regulation, actions, and diseases. Pharmacol Ther.

[5] Braak H, Thal DR, Ghebremedhin E, Del Tredici K (2011). Stages of the pathologic process in Alzheimer disease: age categories from 1 to 100 years. J Neuropathol Exp Neurol.

[6] Breda C, Sathyasaikumar KV, Sograte Idrissi S (2016). Tryptophan-2,3-dioxygenase (TDO) inhibition ameliorates neurodegeneration by modulation of kynurenine pathway metabolites. Proc Natl Acad Sci U S A.

[7] Carlezon WA, Duman RS, Nestler EJ (2005). The many faces of CREB. Trends Neurosci.

[8] Chaikuad A, Tacconi EM, Zimmer J (2014). A unique inhibitor binding site in ERK1/2 is associated with slow binding kinetics. Nat Chem Biol.

[9] Chiti F, Dobson CM (2017). Protein Misfolding, amyloid formation, and human disease: a summary of Progress over the last decade. Annu Rev Biochem.

[10] Cirrito JR, Disabato BM, Restivo JL (2011). Serotonin signaling is associated with lower amyloid-beta levels and plaques in transgenic mice and humans. Proc Natl Acad Sci U S A.

[11] Claeysen S, Bockaert J, Giannoni P (2015). Serotonin: a new Hope in Alzheimer's disease?. ACS Chem Neurosci.

[12] Cooper-Knock J, Kirby J, Ferraiuolo L, Heath PR, Rattray M, Shaw PJ (2012). Gene expression profiling in human neurodegenerative disease. Nat Rev Neurol.

[13] Doyle KM, Kennedy D, Gorman AM, Gupta S, Healy SJ, Samali A (2011). Unfolded proteins and endoplasmic reticulum stress in neurodegenerative disorders. J Cell Mol Med.

[14] Eckman EA, Reed DK, Eckman CB (2001). Degradation of the Alzheimer's amyloid beta peptide by endothelin-converting enzyme. J Biol Chem.

[15] Gartner U, Holzer M, Arendt T (1999). Elevated expression of p21ras is an early event in Alzheimer's disease and precedes neurofibrillary degeneration. Neuroscience.

[16] Gartner U, Holzer M, Heumann R, Arendt T (1995). Induction of p21ras in Alzheimer pathology. Neuroreport.

[17] Good MA, Hale G (2007). The “Swedish” mutation of the amyloid precursor protein (APPswe) dissociates components of object-location memory in aged Tg2576 mice. Behav Neurosci.

[18] Grimm MO, Mett J, Stahlmann CP (2015). APP intracellular domain derived from amyloidogenic beta- and gamma-secretase cleavage regulates neprilysin expression. Front Aging Neurosci.

[19] Grinberg LT, Rub U, Ferretti RE (2009). The dorsal raphe nucleus shows phospho-tau neurofibrillary changes before the transentorhinal region in Alzheimer's disease. A precocious onset?. Neuropathol Appl Neurobiol.

[20] Hanson LR, Fine JM, Svitak AL, Faltesek KA (2013) Intranasal administration of CNS therapeutics to awake mice. J Vis Exp. 10.3791/4440 10.3791/4440 PMC3653240 23608783

[21] Hanson LR, Hafez D, Svitak AL (2011). Intranasal phosphoramidon increases beta-amyloid levels in wild-type and NEP/NEP2-deficient mice. J Mol Neurosci.

[22] Hsiao K, Chapman P, Nilsen S (1996). Correlative memory deficits, Abeta elevation, and amyloid plaques in transgenic mice. Science.

[23] Iijima-Ando K, Iijima K (2010). Transgenic Drosophila models of Alzheimer's disease and tauopathies. Brain Struct Funct.

[24] Jankowska A, Wesolowska A, Pawlowski M, Chlon-Rzepa G (2017). Multi-target-directed ligands affecting serotonergic neurotransmission for Alzheimer's disease therapy: advances in chemical and biological research. Curr Med Chem.

[25] Kakiya N, Saito T, Nilsson P (2012). Cell surface expression of the major amyloid-beta peptide (Abeta)-degrading enzyme, neprilysin, depends on phosphorylation by mitogen-activated protein kinase/extracellular signal-regulated kinase kinase (MEK) and dephosphorylation by protein phosphatase 1a. J Biol Chem.

[26] Kerridge C, Belyaev ND, Nalivaeva NN, Turner AJ (2014). The Abeta-clearance protein transthyretin, like neprilysin, is epigenetically regulated by the amyloid precursor protein intracellular domain. J Neurochem.

[27] Kirouac L, Rajic AJ, Cribbs DH, Padmanabhan J (2017) Activation of Ras-ERK signaling and GSK-3 by amyloid precursor protein and amyloid Beta facilitates neurodegeneration in Alzheimer's disease. eNeuro 4. 10.1523/ENEURO.0149-16.2017 10.1523/ENEURO.0149-16.2017 PMC5367084 28374012

[28] Klein C, Mathis C, Leva G et al. (2015) Gamma-Hydroxybutyrate (Xyrem) ameliorates clinical symptoms and neuropathology in a mouse model of Alzheimer's disease. Neurobiol Aging 36: 832–844 10.1016/j.neurobiolaging.2014.10.003 25457559

[29] Klein C, Patte-Mensah C, Taleb O (2013). The neuroprotector kynurenic acid increases neuronal cell survival through neprilysin induction. Neuropharmacology.

[30] Lovelace MD, Varney B, Sundaram G (2017). Recent evidence for an expanded role of the kynurenine pathway of tryptophan metabolism in neurological diseases. Neuropharmacology.

[31] Maddison DC, Giorgini F (2015). The kynurenine pathway and neurodegenerative disease. Semin Cell Dev Biol.

[32] Musumeci G, Castrogiovanni P, Szychlinska MA (2017). Protective effects of high tryptophan diet on aging-induced passive avoidance impairment and hippocampal apoptosis. Brain Res Bull.

[33] Nalivaeva NN, Beckett C, Belyaev ND, Turner AJ (2012). Are amyloid-degrading enzymes viable therapeutic targets in Alzheimer's disease?. J Neurochem.

[34] Nalivaeva NN, Belyaev ND, Lewis DI (2012). Effect of sodium valproate administration on brain neprilysin expression and memory in rats. J Mol Neurosci.

[35] Nalivaeva NN, Belyaev ND, Turner AJ (2016). New insights into epigenetic and pharmacological regulation of amyloid-degrading enzymes. Neurochem Res.

[36] Nilsson P, Loganathan K, Sekiguchi M (2015). Loss of neprilysin alters protein expression in the brain of Alzheimer's disease model mice. Proteomics.

[37] Nisemblat Y, Belinson H, Dolev I, Michaelson DM (2008). Activation of the amyloid cascade by intracerebroventricular injection of the protease inhibitor phosphoramidon. Neurodegener Dis.

[38] Ognibene E, Middei S, Daniele S (2005). Aspects of spatial memory and behavioral disinhibition in Tg2576 transgenic mice as a model of Alzheimer's disease. Behav Brain Res.

[39] Pacheco-Quinto J, Eckman CB, Eckman EA (2016). Major amyloid-beta-degrading enzymes, endothelin-converting enzyme-2 and neprilysin, are expressed by distinct populations of GABAergic interneurons in hippocampus and neocortex. Neurobiol Aging.

[40] Park MH, Lee JK, Choi S (2013). Recombinant soluble neprilysin reduces amyloid-beta accumulation and improves memory impairment in Alzheimer's disease mice. Brain Res.

[41] Piaceri I, Nacmias B, Sorbi S (2013). Genetics of familial and sporadic Alzheimer's disease. Front Biosci (Elite Ed).

[42] Rosenberg RN, Lambracht-Washington D, Yu G, Xia W (2016). Genomics of Alzheimer disease: a review. JAMA Neurol.

[43] Rub U, Del Tredici K, Schultz C, Thal DR, Braak E, Braak H (2000). The evolution of Alzheimer's disease-related cytoskeletal pathology in the human raphe nuclei. Neuropathol Appl Neurobiol.

[44] Saito T, Iwata N, Tsubuki S (2005). Somatostatin regulates brain amyloid beta peptide Abeta42 through modulation of proteolytic degradation. Nat Med.

[45] Schmid T, Snoek LB, Frohli E, van der Bent ML, Kammenga J, Hajnal A (2015). Systemic regulation of RAS/MAPK signaling by the serotonin metabolite 5-HIAA. PLoS Genet.

[46] Simic G, Babic Leko M, Wray S (2017). Monoaminergic neuropathology in Alzheimer's disease. Prog Neurobiol.

[47] Smith GS, Barrett FS, Joo JH (2017). Molecular imaging of serotonin degeneration in mild cognitive impairment. Neurobiol Dis.

[48] Stroo E, Koopman M, Nollen EA, Mata-Cabana A (2017). Cellular regulation of amyloid formation in aging and disease. Front Neurosci.

[49] Tentolouris-Piperas V, Ryan NS, Thomas DL, Kinnunen KM (2017). Brain imaging evidence of early involvement of subcortical regions in familial and sporadic Alzheimer's disease. Brain Res.

[50] Vermeiren Y, Van Dam D, Aerts T, Engelborghs S, De Deyn PP (2014). Monoaminergic neurotransmitter alterations in postmortem brain regions of depressed and aggressive patients with Alzheimer's disease. Neurobiol Aging.

[51] Wang Z, Zhang XJ, Li T, Li J, Tang Y, Le W (2014). Valproic acid reduces neuritic plaque formation and improves learning deficits in APP(Swe) /PS1(A246E) transgenic mice via preventing the prenatal hypoxia-induced down-regulation of neprilysin. CNS Neurosci Ther.

[52] Weiner MW, Veitch DP, al APS (2017). Recent publications from the Alzheimer's disease neuroimaging initiative: reviewing progress toward improved AD clinical trials. Alzheimer's Dement.

[53] Wendt G, Kemmel V, Patte-Mensah C (2014). Gamma-hydroxybutyrate, acting through an anti-apoptotic mechanism, protects native and amyloid-precursor-protein-transfected neuroblastoma cells against oxidative stress-induced death. Neuroscience.

[54] Westerman MA, Cooper-Blacketer D, Mariash A (2002). The relationship between Abeta and memory in the Tg2576 mouse model of Alzheimer's disease. J Neurosci.

[55] Whitehouse IJ, Brown D, Baybutt H (2016). Ablation of prion protein in wild type human amyloid precursor protein (APP) transgenic mice does not Alter the proteolysis of APP, levels of amyloid-beta or pathologic phenotype. PLoS One.

[56] Whyte LS, Lau AA, Hemsley KM, Hopwood JJ, Sargeant TJ (2017). Endo-lysosomal and autophagic dysfunction: a driving factor in Alzheimer's disease?. J Neurochem.

[57] Yassine N, Lazaris A, Dorner-Ciossek C (2013). Detecting spatial memory deficits beyond blindness in tg2576 Alzheimer mice. Neurobiol Aging.

